# An Adaptation of Wintering Water Birds to Man-Made Weirs in Relation to the Freeze–Thaw Process in Tancheon Stream, Korea

**DOI:** 10.3390/ani15213161

**Published:** 2025-10-30

**Authors:** Jangho Lee, Chan-Ryul Park, Hong-Duck Sou

**Affiliations:** 1 Natural Environment Research Division, National Institute of Environmental Research, Incheon 22689, Republic of Korea; ficedula01@korea.kr; 2 Livable Urban Forests Research Center, National Institute of Forest Science, Seoul 02455, Republic of Korea; hongducksou@korea.kr

**Keywords:** freeze–thaw pattern, weir impact, water bird distribution, urban streams

## Abstract

**Simple Summary:**

Man-made weirs control summer flooding, but also provide diverse habitats for wintering water birds through the freeze–thaw process in urban streams.

**Abstract:**

The roles of man-made weirs in flood control during the summer monsoon are well-known, but the function of habitats for wintering water birds has not been explored. This study examined the effects of man-made weirs on the wintering distribution of water birds in Tancheon Stream, Korea, with a focus on the impact of the freeze–thaw process. Data collected from January to February 2003 included thawed water surface ratios, water depths, sandbar areas, and bird distribution under different weather conditions. The analysis revealed a strong positive correlation between thawed surface areas in front of the weir’s drop structures and the abundance of dabbling and diving water birds during severe cold conditions with temperatures below −9 °C. However, it was also observed that the stagnant water impounded by the weirs tended to freeze easily in winter, making it difficult for water birds to inhabit those areas. These results suggest an adaptation of wintering water birds to man-made weirs in urban streams, providing significant insights for enhancing habitat functions in urban stream management.

## 1. Introduction

In cities worldwide, several urban streams have been modified by the installation of impervious surfaces and channelized through the construction of embankments, concrete or rubber weirs, and drop structures. These structures aim to reduce flooding hazards and control water flow [1,2,3,4]. However, these changes are known to negatively impact water birds, primarily due to the reduction in natural habitats such as sandbars and the decline in fish diversity, which serves as prey for the birds [5,6,7]. In contrast, positive effects have been reported for the taxa of large-bodied emerging insects, total bird abundance, and cowbird parasitism during the breeding season [8]. In cities, urbanization and development affect a variety of behavioral patterns. Many birds appeared to favor developed shorelines; however, their responses differed between summer and winter, exhibiting distinct behaviors [9]. There are debates about the urbanization effect on mobile birds, as well as the impact of urban tolerance on the different reactions of avoidance and preference exhibited by birds worldwide [10].

Every winter, over 900,000 water birds migrate from the Northern Hemisphere to South Korea, with approximately 40,000 overwintering in the Hangang River watershed, which flows through Seoul, the capital of South Korea [11,12]. During severe winter weather, water surfaces in this watershed frequently freeze, causing birds to relocate to non-frozen areas [13,14]. Tributaries of the Hangang River are particularly susceptible to freezing due to reduced water flow, impervious surfaces, and channelization [15]. Weirs in urban streams can exacerbate freezing by creating conditions similar to reservoir dams, leading to stagnant water that freezes easily. Thus, the freeze–thaw dynamics created by weirs could significantly affect the distribution of water birds in urban streams during winter.

Notably, man-made weirs may cause water surfaces to provide wintering water birds with forage prey during normal winter weather. However, the freezing of water surfaces during severe winter weather may be detrimental for wintering water birds by reducing the available habitat for them.

In 2002, the Seongnam City Tancheon Development Plan aimed to restore the Tancheon to an ecological river, improve the surrounding landscape, and create a bicycle path connecting the Tancheon to the Han River. The plan was implemented in several phases beginning in 2002 and has contributed to improving the water quality of the Tancheon, developing an eco-friendly riverbed, and creating a pleasant recreational space for residents [16,17,18].

However, several cities adopted the free-flowing river system and removed man-made weirs since the 2000s in Korea. Extreme weather, including low precipitation and harsh winters, can cause severe droughts, negatively affecting wintering habitat conditions for migratory water birds, particularly in the tributaries of the Han River. Therefore, the distribution of water birds before the removal of the weirs can provide valuable guidance for winter habitat management under current meteorological conditions in Korea. To ensure the effects of man-made weirs, it is essential to investigate their impacts on their distribution in relation to freeze–thaw dynamics. Therefore, this study aimed to assess the effects of weirs on the distribution of water birds at Tancheon Stream using a dataset from 2003 in South Korea. Specifically, we examined correlations between the spatial distribution of water birds and environmental factors, such as thawed water surface areas, sandbars, and water depths. This data will provide insights into the value of weirs in urban environments during extreme weather conditions.

## 2. Materials and Methods

### 2.1. Study Area

**Figure 1:**
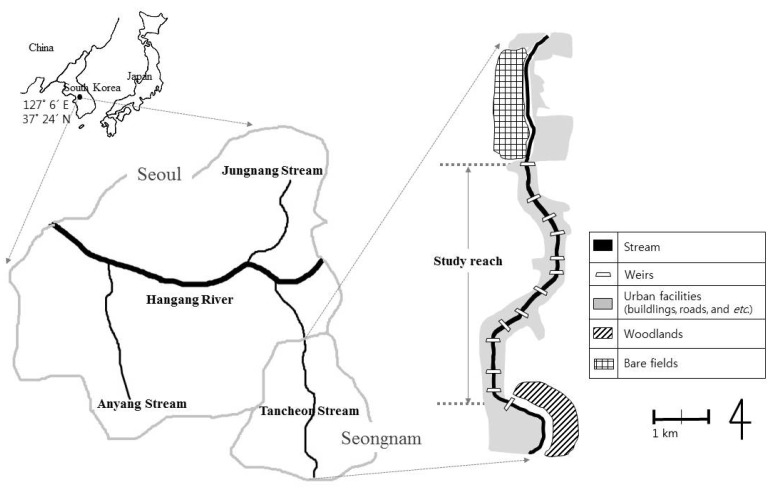
Location of study area along the Tancheon Stream, Seongnam City, Korea.

We conducted the study in Tancheon Stream, which flows through Seongnam City, Korea (Figure 1). The base channel of the stream was lined with concrete, and 17 weirs were distributed along its length. The middle reach of the stream, which spanned approximately 8.4 km with a channel width of about 50 m, was the focus of this study. This section comprised 13 weirs, each measuring approximately 50 m in length and 1–4 m in height. The surrounding land use was primarily urban.

**Figure 2:**
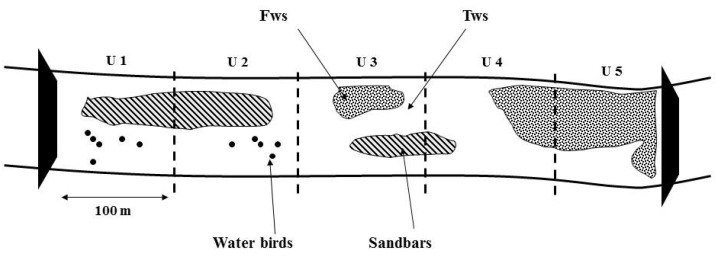
Schematic explanation of survey sites in this study (U: unit; Fws: frozen area of water surface; Tws: thawed area of water surface).

The study reach was divided into 76 units, each approximately 100 m apart, covering the distance between weirs (Figure 2). Of these, only 52 units were included in the analysis, excluding 24 units that contained bridges. The 52 units were categorized based on their distance from the weirs, as follows: 0–100 m (11 units), 100–200 m (six units), 200–300 m (10 units), 300–400 m (four units), 400–500 m (10 units), 500–600 m (six units), and 600–700 m (five units).

### 2.2. Collected Data and Measured Parameters

We collected the number of water birds, and we measured the parameters of water depths, thawed ratio, temperature, distance from weirs, and area of sandbars.

We recorded the number of water birds in the stream channel during the first two hours after sunrise on the survey days. Binoculars (8 × 30 magnification) were used to identify birds, with reference to a field guide [19]. Also, we divided the observed birds into diving ducks and dabbling ducks to comprehend the habitat use of water depth at the study areas (Table 1).

**Figure 3:**
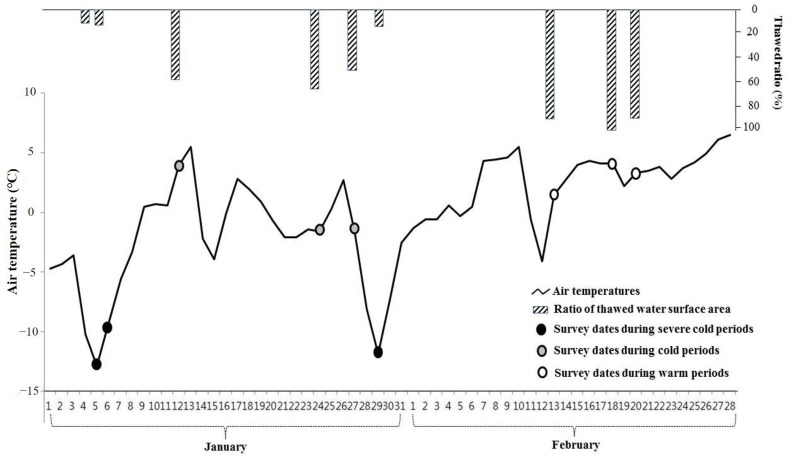
The distributions of air temperatures and thawed water surface ratios in the study reach of Tancheon during the survey periods (5 January–20 February 2003).

To analyze the impact of the freeze–thaw phenomenon on water birds’ distributions, we conducted our survey during the stable wintering season from January to February 2003. The survey days (January 5, 6, 12, 24, 27, 29, and February 13, 18, 20) were categorized based on weather severity—severe cold days (mean thawed water surface ratio < 20%, mean air temperature < −9 °C), cold days (thawed ratio 50–70%, air temperature −2 to 4 °C), and warm days (thawed ratio > 90%, air temperature 0–5 °C) (Figure 3). Air temperature data were obtained from the Korea Meteorological Administration [20].

For water depths, measurements were taken under the assumption that depths remained constant, given the low precipitation during the survey period (32.5 mm of the annual total yearly precipitation of 2012 mm [20]. In each unit (approximately 100 m), four transect lines were established at 20 m intervals across the stream. Each transect had five measurement points (5, 10, 20, 30, and 40 m from the right bank to the left). The area of sandbars was assumed to remain constant in the same way as the water depth. Using a 1:1500 digital topographic map provided by Seongnam City [18], the area for each unit was calculated with AutoCAD Release 14. To estimate the thawed water surface areas, the thawed regions were sketched onto the survey map at a scale of 1:1500 during each survey day. Next, the map was overlaid with a translucent acetate grid divided into squares of 0.25 cm^2^ each. We then counted the number of grid squares (interstices) that fell completely within the thawed area, assigning a value of 1 to each. Interstices that landed precisely on the boundary of the thawed area were given a value of 0.5 per unit. The total count was multiplied by 56.25 m^2^ to calculate the thawed water surface area in square meters. Finally, the thawed ratio (%) was calculated by comparing the thawed area to the total area of each unit.

### 2.3. Statistical Analysis

The measured values are represented in the graph as means and standard error (standard deviation divided by the square root of the number of samples). To examine the effects of weirs on the distribution of water birds in relation to the freeze–thaw phenomenon, we analyzed the statistical correlations between water bird distributions and environmental variables. Statistical analysis was performed using the statistical software R 4.2.0 [21].

## 3. Results

### 3.1. Abundance of the Observed Water Birds

Among the observed water birds, the abundance was highest in the order of Spot-billed Duck (*Anas poecilorhyncha*), Little Grebe (*Tachybaptus ruficollis*), and Common Merganser (*Mergus merganser*) (Table 1).

### 3.2. Distribution of Environmental Variables

**Figure 4:**
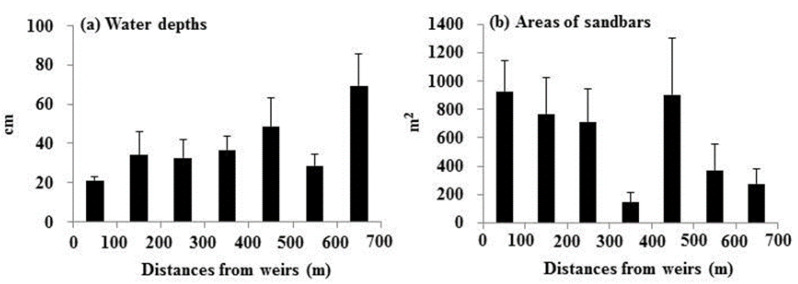
Distributions of the water depths and the sandbar areas in relation to distance from weirs (mean ± S.E.) at each distance; 0–100 m (11 samples), 100–200 m (six samples), 200–300 m (10 samples), 300–400 m (four samples), 400–500 m (10 samples), 500–600 m (six samples), and 600–700 m (five samples).

The mean water depths (ranging from 21 to 69 cm) increased gradually with distance from the weirs (Figure 4a, Table S1), showing a statistically significant correlation with the distance (r = 0.61, *p* < 0.01). In contrast, the mean areas of sandbars were primarily concentrated in the 0–300 m and 400–500 m sections, while the 300–400 m and 500–700 m sections showed relatively smaller sandbar areas (Figure 4b). The correlation with the distance was statistically significant (r = −0.61, *p* < 0.01).

**Figure 5:**
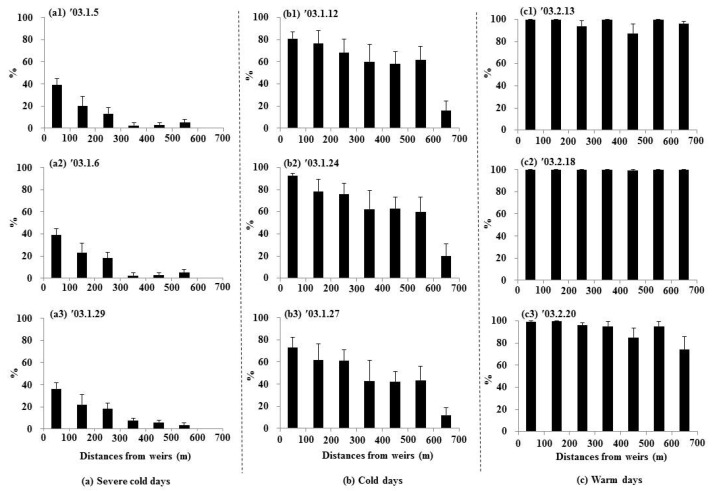
Thawed ratios of the water surfaces in relation to distance from weirs (mean ± S.E.) at each distance; 0–100 m (11 samples), 100–200 m (six samples), 200–300 m (10 samples), 300–400 m (four samples), 400–500 m (10 samples), 500–600 m (six samples), and 600–700 m (five samples).

During severe cold weather conditions, the highest mean thawed ratios were observed at the front of the weirs (0–100 m), ranging from 36% to 39% (Figure 5(a1–a3)), Table S2). In the subsequent sections (100–200 m, 200–300 m), thawed ratios decreased to 20–23% and 13–18%, respectively. Other sections (300–700 m) exhibited lower thawed ratios of less than 8%. The correlation with distance was statistically significant (r = −0.90, *p* < 0.001). Under the cold weather conditions, the front section of the weirs (0–100 m) exhibited higher thawed ratios (73–93%) compared to the severe cold conditions (Figure 5(b1–b3)). The mean thawed ratios gradually decreased with distance from the weirs, and the correlation with distance remained statistically significant (r = −0.85, *p* < 0.001). However, during the warmer conditions, all sections displayed higher thawed ratios (74–100%) compared to the cold conditions (Figure 5(c1–c3)). However, the correlation with distance was not statistically significant (r = −0.37).

Water depth showed a statistically significant negative correlation with the thawed ratio (r = −0.75, *p* < 0.001) during the severe cold weather conditions. During cold weather conditions, the correlation coefficient slightly decreased to r = −0.67 (*p* < 0.001). During the warm weather period when most survey units were thawed, the relationship between water depth and thawed ratio was not statistically significant (r = −0.38). The area of sandbars showed a statistically significant positive correlation with the thawed ratio (r = 0.70, *p* < 0.001) during severe cold conditions. Under cold conditions, the correlation coefficient decreased to r = 0.60 (*p* < 0.01). During warm conditions, when the average thawed ratios across all sections exceeded about 74%, the correlation between sandbar area and thawed ratio was not statistically significant (r = 0.013).

### 3.3. Distribution of Water Birds

**Figure 6:**
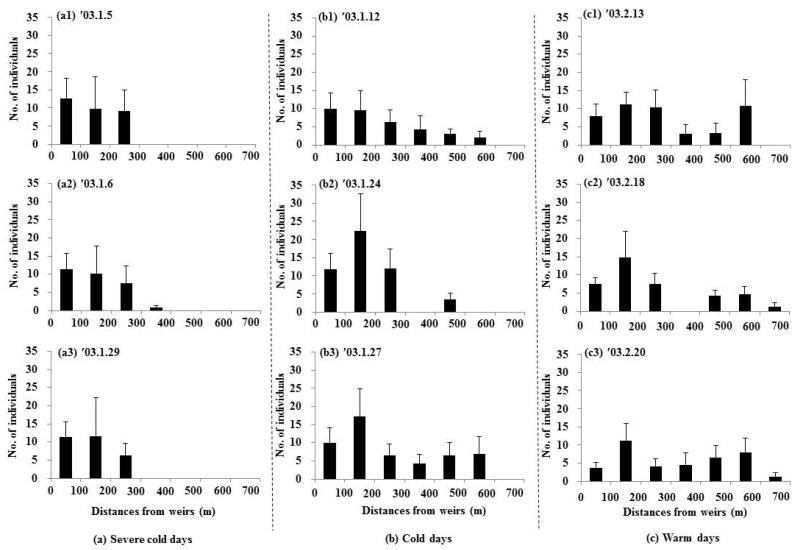
Distributions of the dabbling duck abundance in relation to distance from weirs (mean ± S.E.) at each distance; 0–100 m (11 samples), 100–200 m (six samples), 200–300 m (10 samples), 300–400 m (four samples), 400–500 m (10 samples), 500–600 m (six samples), and 600–700 m (five samples).

**Figure 7:**
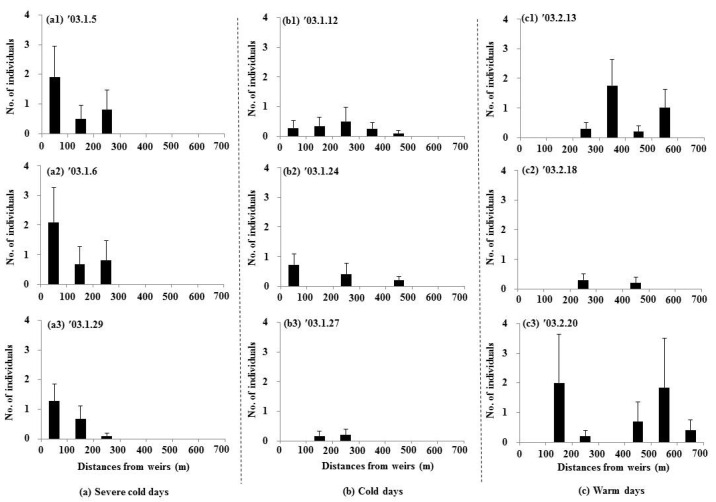
Distributions of the diving water birds’ abundance in relation to distance from weirs (mean ± S.E.) at each distance; 0–100 m (11 samples), 100–200 m (six samples), 200–300 m (10 samples), 300–400 m (four samples), 400–500 m (10 samples), 500–600 m (six samples), and 600–700 m (five samples).

During severe cold weather conditions, dabbling ducks and diving water birds were densely distributed within sections close to the weirs (0–300 m) (Figure 6(a1–a3) and Figure 7(a1–a3), Tables S3 and S4). As the weather transitioned to cold conditions, the birds spread out over a wider area (dabbling ducks: up to 600 m, diving water birds: up to 500 m) (Figure 6(b1–b3) and Figure 7(b1–b3)). Under warmer conditions, dabbling ducks tended to spread up to 700 m from the weirs (Figure 6(c1–c3)). Notably, diving water birds were not observed in the sections near the weirs (0–100 m) during warmer days, likely due to the shallow water depth in these areas (Figure 7(c1–c3)).

To further examine the relationships between the abundance of water birds and environmental variables, we calculated correlation coefficients (Table 2).

During severe cold weather, the abundance of both dabbling ducks and diving water birds showed strong positive correlations with the thawed water surface ratios (dabbling ducks: r = 0.85; diving water birds: r = 0.87). However, these correlations gradually weakened as the weather warmed (cold days: dabbling ducks (r = 0.68), diving water birds (r = 0.60); warm days: dabbling ducks (r = 0.29), diving water birds (r = −0.27)). The abundance of dabbling ducks was also positively and significantly correlated with the area of sandbars across all periods (severe cold days: r = 0.58; cold days: r = 0.62; warm days: r = 0.44). In contrast, the abundance of diving water birds showed a significant positive correlation with sandbar areas only during the severe cold days (r = 0.64). Water depth had a negative and significant correlation with the abundance of dabbling ducks during all periods (severe cold days: r = −0.59; cold days: r = −0.55; warm days: r = −0.55). For diving water birds, a significant negative correlation with water depth was observed only during severe cold days (r = −0.65).

## 4. Discussion

The results of this study highlight the significant role of weirs in shaping the winter distribution of water birds in urban streams, specifically in the Tancheon Stream. Our observations underline that freeze–thaw dynamics created by the presence of weirs influence habitat availability for water birds, with a particularly strong impact during severe winter weather conditions.

Assuming that the warm period, when most of the water surface was thawed, had minimal influence on bird distribution due to ice formation, dabbling ducks in this study were primarily found in areas with abundant sandbars and shallow water. In contrast, diving ducks showed little correlation with sandbar area or water depth. As temperatures dropped and the frozen surface area increased, the distribution patterns of water birds changed. Dabbling ducks became more concentrated near the weirs, where water remained thawed, typically characterized by shallow depths and extensive sandbars. During the severe cold period, despite the shallow water, diving ducks were also compelled to concentrate near the weirs, likely due to the availability of thawed water.

The presence of weirs appears to provide a refuge for water birds during severe cold weather, as thawed water surface areas were predominantly found in front of the weir’s drop structures even in temperatures below −9 °C. These thawed areas likely offer critical feeding grounds when other sections of the stream are frozen. Our correlation analysis supports this, showing a strong positive association between thawed water surface ratios and water bird abundance, particularly for dabbling ducks and diving water birds during severe cold conditions. In these areas, water birds are presumed to engage primarily in resting and feeding behaviors. Lee [4] analyzed the diurnal behaviors of wintering ducks in Tancheon Stream during the winters of 1999 to 2000. The study categorized behaviors into feeding, resting and sleeping, movement, alertness, etc. The results indicated that Spot-billed Ducks predominantly exhibited resting (50.05%) and feeding (13.65%) behaviors. Common Teal showed a dominance of resting (42.37%) and feeding (41.65%), while Mallards were primarily involved in resting (36.30%) and movement (21.92%), with resting behavior being a common dominant activity among the three species.

The presence of abundant sandbars in the shallow areas near the weirs likely influenced the distribution of dabbling ducks, alongside the thawed water surfaces. The formation of weirs, as artificial structures, can impact water depth and river flow, thereby affecting sandbar formation [22,23], which in turn can influence the distribution of water birds [24,25]. However, the areas where water is impounded by the weir tend to have deeper depths and stagnant conditions, which make them more susceptible to freezing. This compels water birds to gather in the confined thawed areas near the weir’s drop structure. As habitat space becomes limited, the risk of exposure to disturbances from humans and other sources may increase [26,27,28]. Lee [4] investigated disturbances impacting the feeding and resting behavior of water birds in Tancheon Stream. Human activity accounted for 86% of the disturbances, followed by terrestrial animals like dogs and cats (6.2%), aircraft (4.7%), and raptors (1.6%). Additionally, vehicle noise was suggested as a potential disturbance factor. These disturbances are likely to have a stronger impact on water birds concentrated in limited areas during periods of severe freezing.

This study could be a case study of wintering water birds from January to February in 2003, and a short snapshot of the urban stream could give a bias to the restoration project of the river and stream in a city. However, the ecological importance of man-made weir function suggests the countermeasure to maintain the water depth for wintering water birds during the extreme drought and flood weather event in recent at a city.

The findings from our study suggest several implications for urban stream management and the conservation of water birds. To ensure stable wintering conditions for water birds, urban planners and conservationists should consider the role of weirs in maintaining thawed water surfaces in front of weir drop structures during winter. However, it is equally important to mitigate the negative impacts of weirs, such as the potential for excessive freezing in stagnant waters. Management strategies could include partial removal or modification of weirs to promote flow variability, creating more dynamic and ecologically diverse habitats [29,30].

## 5. Conclusions

This study examined the impact of weirs, a characteristic feature of urban streams in South Korea, on winter freeze–thaw dynamics and the distribution of water birds. It highlighted both the positive and negative effects of water bird distribution associated with weir structures, emphasizing the need for improvements in the design and management of weirs. The implication of this study will be an alternative policy to maintain the water depth in the era of extreme climate such as drought and flood in a city. So, the adjustment and the structural change in man-made weirs would be a proper policy rather than the simple removal of man-made weirs in a stream. Future studies should consider a broader range of environmental factors, such as human disturbances, to gain a more comprehensive understanding of how urbanization influences water bird ecology. Additionally, monitoring over multiple winter seasons would help capture year-to-year variability and refine conservation recommendations for managing urban water bodies effectively.

## Figures and Tables

**Table 1:** Abundance of the water birds in the study area (*n* = 9).

Common Name	Scientific Name	Mean	S.E.	Min.	Max.
Spot-billed Duck ^1^	*Anas poecilorhyncha*	310.3	18.9	256.0	399.0
Little Grebe ^2^	*Tachybaptus ruficollis*	11.2	3.7	0.0	28.0
Common Merganser ^2^	*Mergus merganser*	6.3	2.5	1.0	24.0
Common Teal ^1^	*Anas crecca*	5.6	1.3	1.0	11.0
Mallard ^1^	*Anas platyrhynchos*	5.4	1.4	0.0	14.0
Little Egret	*Egretta garzetta*	3.2	1.0	0.0	8.0
Grey Heron	*Ardea cinerea*	1.4	0.4	0.0	3.0
Pochard ^2^	*Aythya ferina*	1.2	0.5	0.0	4.0
Herring Gull	*Larus argentatus*	0.7	0.2	0.0	1.0
Black-backed Wagtail	*Motacilla lugens*	0.6	0.3	0.0	2.0
Pintail ^1^	*Anas acuta*	0.3	0.2	0.0	1.0
Black-crowned Night Heron	*Nycticorax nycticorax*	0.2	0.2	0.0	2.0

^1^: dabbling ducks, ^2^: diving water birds.

**Table 2:** Correlations between environmental variables and the abundance of water birds.

Environmental Variables	No. of Individuals					
	Severe Cold Days (*n* = 21)		Cold Days (*n* = 21)		Warm Days (*n* = 21)	
	Dabbling ^1^	Diving ^2^	Dabbling	Diving	Dabbling	Diving
Thawed ratios (%)	0.85 **	0.87 **	0.68 **	0.60 **	0.29 ^ns^	−0.27 ^ns^
Areas of sandbars (m^2^)	0.58 **	0.64 **	0.62 **	0.39 ^ns^	0.44 *	−0.10 ^ns^
Water depths (cm)	−0.59 **	−0.65 **	−0.55 **	−0.32 ^ns^	−0.55 *	0.07 ^ns^

^1^: dabbling ducks, ^2^: diving water birds, ^ns^: non-significant, *: *p*-value < 0.05, **: *p*-value < 0.01.

## Data Availability

The original contributions presented in this study are included in the article. Further inquiries can be directed to the corresponding author(s).

## Supplementary Materials

The following supporting information can be downloaded at: https://www.mdpi.com/article/10.3390/ani15213161/s1, Table S1: Water depths and sandbar areas in relation to distance from weirs in Tancheon, 2003; Table S2: Thawed ratios (%) of the water surfaces in relation to distance from weirs in Tancheon, 2003; Table S3: Abundance of the dabbling ducks in relation to distance from weirs in Tancheon, 2003; Table S4: Abundance of the diving water birds in relation to distance from weirs in Tancheon, 2003.

## Abbreviations

The following abbreviations are used in this manuscript:

Fws	Frozen area of water surface
Tws	Thawed area of water surface
