# Supplementary material for: An Adaptation of Wintering Water Birds to Man-Made Weirs in Relation to the Freeze–Thaw Process in Tancheon Stream, Korea
Source: Animals (Basel). 2025 Oct 30;15(21):3161. doi: 10.3390/ani15213161 (PMC12610298; doi:10.3390/ani15213161)
Supplement: Supplementary file 1 [file animals-15-03161-s001.zip › animals-3938051-supplementary.pdf]

Table S1. Water depths and sandbar areas in relation to distance from weirs in Tancheon, 2003

Variables		Distance (m)						
		0-100 (n=11)	100-200 (n=6)	200-300 (n=10)	300-400 (n=4)	400-500 (n=10)	500-600 (n=6)	600-700 (n=5)
Water depth (cm)	Mean	21	34	33	36	49	29	69
	S.E.	2	12	9	7	15	6	16
	Min.	13	17	15	16	16	16	22
	Max.	31	100	119	50	177	58	110
Areas of sandbars (m^2)	Mean	929	764	710	142	903	365	274
	S.E.	215	265	235	71	401	193	106
	Min.	186	0	0	0	0	0	0
	Max.	2,729	1,712	2,174	305	4,488	1,251	562

Table S2. Thawed ratios (%) of the water surfaces in relation to distance from weirs in Tancheon, 2003

Distance (m)		Severe cold days			Cold days			Warm days		
		Jan. 5	Jan. 6	Jan. 29	Jan. 12	Jan. 24	Jan. 27	Feb. 13	Feb.18	Feb. 20
0-100 (n=11)	Mean	39.1	39.1	36.4	80.9	92.7	72.7	100.0	100.0	99.1
	S.E.	5.5	5.5	5.6	6.0	1.9	9.4	0.0	0.0	0.9
	Min.	10	10	10	40	80	30	100	100	90
	Max.	70	70	60	100	100	100	100	100	100
100-200 (n=6)	Mean	20.0	23.3	21.7	76.7	78.3	61.7	100.0	100.0	100.0
	S.E.	8.5	8.4	9.3	11.7	10.9	14.4	0.0	0.0	0.0
	Min.	0	0	0	20	20	0	100	100	100
	Max.	60	60	50	100	100	100	100	100	100
200-300 (n=10)	Mean	13.0	18.0	18.0	68.0	76.0	61.0	94.0	100.0	96.0
	S.E.	5.8	5.3	5.6	12.1	9.5	9.6	4.7	0.0	2.1
	Min.	0	0	0	0	0	0	50	100	80
	Max.	60	60	50	100	100	100	100	100	100
300-400 (n=4)	Mean	2.5	2.5	8	60.0	62.5	42.5	100.0	100.0	95.0
	S.E.	2.2	2.2	2.2	15.8	16.7	18.8	0.0	0.0	4.3
	Min.	0	0	0	20	20	0	100	100	80
	Max.	10	10	10	100	100	100	100	100	100
400-500 (n=10)	Mean	3.0	3.0	6.0	58.0	63.0	42.0	87.0	99.0	85.0
	S.E.	2.0	2.0	2.1	10.8	10.5	9.4	8.6	0.9	8.7
	Min.	0	0	0	0	0	0	10	90	30
	Max.	20	20	20	100	100	90	100	100	100
500-600 (n=6)	Mean	5.0	5.0	3.3	61.7	60.0	43.3	100.0	100.0	95.0
	S.E.	3.1	3.1	1.9	12.3	13.1	12.8	0.0	0.0	4.6
	Min.	0	0	0	0	0	0	100	100	70
	Max.	20	20	10	90	100	80	100	100	100
600-700 (n=5)	Mean	0.0	0.0	0.0	16.0	20.0	12.0	96.0	100.0	74.0
	S.E.	0.0	0.0	0.0	8.8	11.3	6.6	2.2	0.0	11.9
	Min.	0	0	0	0	0	0	90	100	30
	Max.	0	0	0	40	60	30	100	100	100

Table S3. Abundance of the dabbling ducks in relation to distance from weirs in Tancheon, 2003

Distance (m)		Severe cold days			Cold days			Warm days		
		Jan. 5	Jan. 6	Jan. 29	Jan. 12	Jan. 24	Jan. 27	Feb. 13	Feb.18	Feb. 20
0-100 (n=11)	Mean	12.6	11.4	11.4	9.9	11.8	9.9	7.9	7.5	3.7
	S.E.	5.6	4.5	4.1	4.4	4.3	4.2	3.4	1.8	1.5
	Min.	0	0	0	0	0	0	0	0	0
	Max.	52	38	42	43	47	49	41	19	14
100-200 (n=6)	Mean	9.7	10.2	11.7	9.5	22.3	17.3	11.2	14.8	11.2
	S.E.	8.8	7.5	10.7	5.5	10.1	7.6	3.4	7.2	4.9
	Min.	0	0	0	0	0	0	0	0	0
	Max.	58	51	70	38	69	57	22	52	34
200-300 (n=10)	Mean	9.1	7.5	6.4	6.3	11.9	6.6	10.4	7.6	4.1
	S.E.	5.9	4.9	3.3	3.3	5.4	3.0	4.8	2.9	2.1
	Min.	0	0	0	0	0	0	0	0	0
	Max.	55	45	31	30	54	29	47	30	20
300-400 (n=4)	Mean	0.0	0.8	0.0	4.3	0.0	4.3	3.0	0.0	4.5
	S.E.	0.0	0.6	0.0	3.7	0.0	2.7	2.6	0.0	3.3
	Min.	0	0	0	0	0	0	0	0	0
	Max.	0	3	0	17	0	13	12	0	16
400-500 (n=10)	Mean	0.0	0.0	0.0	3.1	3.5	6.5	3.2	4.3	6.5
	S.E.	0.0	0.0	0.0	1.3	1.7	3.5	2.8	1.5	3.3
	Min.	0	0	0	0	0	0	0	0	0
	Max.	0	0	0	12	13	35	30	14	32
500-600 (n=6)	Mean	0.0	0.0	0.0	2.0	0.0	6.8	10.7	4.7	7.8
	S.E.	0.0	0.0	0.0	1.8	0.0	4.9	7.3	2.1	4.0
	Min.	0	0	0	0	0	0	0	0	0
	Max.	0	0	0	12	0	33	49	15	27
600-700 (n=5)	Mean	0.0	0.0	0.0	0.0	0.0	0.0	0.0	1.2	1.2
	S.E.	0.0	0.0	0.0	0.0	0.0	0.0	0.0	1.1	1.1
	Min.	0	0	0	0	0	0	0	0	0
	Max.	0	0	0	0	0	0	0	6	6

Table S4. Abundance of the diving water birds in relation to distance from weirs in Tancheon, 2003

Distance (m)		Severe cold days			Cold days			Warm days		
		Jan. 5	Jan. 6	Jan. 29	Jan. 12	Jan. 24	Jan. 27	Feb. 13	Feb.18	Feb. 20
0-100 (n=11)	Mean	1.9	2.1	1.3	0.3	0.7	0.0	0.0	0.0	0.0
	S.E.	1.0	1.2	0.6	0.3	0.4	0.0	0.0	0.0	0.0
	Min.	0	0	0	0	0	0	0	0	0
	Max.	10	12	6	3	3	0	0	0	0
100-200 (n=6)	Mean	0.5	0.7	0.7	0.3	0.0	0.2	0.0	0.0	2.0
	S.E.	0.5	0.6	0.5	0.3	0.0	0.2	0.0	0.0	1.6
	Min.	0	0	0	0	0	0	0	0	0
	Max.	3	4	3	2	0	1	0	0	11
200-300 (n=10)	Mean	0.8	0.8	0.1	0.5	0.4	0.2	0.3	0.3	0.2
	S.E.	0.7	0.7	0.1	0.5	0.4	0.2	0.2	0.2	0.2
	Min.	0	0	0	0	0	0	0	0	0
	Max.	7	7	1	5	4	2	2	2	2
300-400 (n=4)	Mean	0.0	0.0	0.0	0.3	0.0	0.0	1.8	0.0	0.0
	S.E.	0.0	0.0	0.0	0.2	0.0	0.0	0.9	0.0	0.0
	Min.	0	0	0	0	0	0	0	0	0
	Max.	0	0	0	1	0	0	4	0	0
400-500 (n=10)	Mean	0.0	0.0	0.0	0.1	0.2	0.0	0.2	0.2	0.7
	S.E.	0.0	0.0	0.0	0.1	0.1	0.0	0.2	0.2	0.7
	Min.	0	0	0	0	0	0	0	0	0
	Max.	0	0	0	1	1	0	2	2	7
500-600 (n=6)	Mean	0.0	0.0	0.0	0.0	0.0	0.0	1.0	0.0	1.8
	S.E.	0.0	0.0	0.0	0.0	0.0	0.0	0.6	0.0	1.7
	Min.	0	0	0	0	0	0	0	0	0
	Max.	0	0	0	0	0	0	4	0	11
600-700 (n=5)	Mean	0.0	0.0	0.0	0.0	0.0	0.0	0.0	0.0	0.4
	S.E.	0.0	0.0	0.0	0.0	0.0	0.0	0.0	0.0	0.4
	Min.	0	0	0	0	0	0	0	0	0
	Max.	0	0	0	0	0	0	0	0	2
